# Ulk1 over-expression in human gastric cancer is correlated with patients' T classification and cancer relapse

**DOI:** 10.18632/oncotarget.16734

**Published:** 2017-03-31

**Authors:** Min-Bin Chen, Xiao-Zhi Ji, Yuan-yuan Liu, Ping Zeng, Xin-Yu Xu, Rong Ma, Zheng-Dong Guo, Jian-Wei Lu, Ji-Feng Feng

**Affiliations:** ^1^ Department of Radiotherapy & Oncology, Kunshan First People's Hospital Affiliated to Jiangsu University, Kunshan, Jiangsu Province, China; ^2^ Departments of Medical Oncology, Jiangsu Cancer Hospital Affiliated to Nanjing Medical University, Jiangsu Province Institute of Cancer, Nanjing, Jiangsu Province, China; ^3^ Xuzhou Medical University, Xuzhou, Jiangsu Province, China; ^4^ Departments of Pathology, Jiangsu Cancer Hospital Affiliated to Nanjing Medical University, Jiangsu Province Institute of Cancer, Nanjing, Jiangsu Province, China; ^5^ Clinical Cancer Research Center, Jiangsu Cancer Hospital Affiliated to Nanjing Medical University, Nanjing, Jiangsu Province, China

**Keywords:** gastric cancer, Ulk1, survival, T classification, cancer relapse

## Abstract

Ulk1 is a key autophagy protein. Here, we tested expression and potential function of Ulk1 in human gastric cancer. *Ulk1* mRNA and protein were significantly elevated in multiple fresh human gastric cancer tissues. Its level was relatively low in surrounding normal epithelial tissues. Ulk1 over-expression was also observed in several gastric cancer cell lines (AGS, HGC-27, and SNU601). Remarkably, Ulk1 knockdown by targeted-shRNA inhibited AGS gastric cancer cell survival and proliferation. On the other hand, exogenous Ulk1 over-expression could further promote AGS cell survival and proliferation. Immunohistochemistry (IHC) staining assay of 145 paraffin-embedded gastric cancer tissues showed that Ulk1 was over-expressed in majority (114 out of 145) of gastric cancer tissues. Importantly, high Ulk1 expression in gastric cancer was correlated with patients’ T classification and cancer relapse. Together, we demonstrate that Ulk1 over-expression in human gastric cancer is pro-survival. Its over-expression is associated with patients’ T classification and cancer relapse.

## INTRODUCTION

Gastric cancer is a major health threat in China and other countries [1–3]. Although significant progresses have been made in pathological mechanism research and therapeutic strategies for this devastating disease, the prognosis is still poor [4–6]. Its incidence, on the other hand, has been steadily rising in China [7] and possible other countries [1, 8]. The application of the conventional cytotoxic agents and molecular-targeted agents is not satisfactory when facing pre-existing and/or acquired resistance [5, 6, 9]. Therefore, research focus is to understand the molecular mechanisms of gastric cancer tumorigenesis and progression, and to identify novel oncotarget proteins for this malignancy [5, 6, 9].

Autophagy is important in the progression of a number of cancers [10–15]. Although some studies suggested that autophagy might promote cancer cell death, many others have proposed that autophagy is indeed important for cancer cell survival and apoptosis-resistance [13, 14, 16, 17]. Among all the autophagy-related genes (ATGs), Ulk1 (UNC51-like kinase 1) serves as a key upstream signaling for autophagy initiation [18–21]. This serine-threonine kinase is the mammalian orthologue of yeast *ATG1* [18–21]. Ulk1 forms a complex with Atg13, FIP200, and Atg101, which is required to start autophagy [18–21]. It expression and potential functions in human gastric cancer were tested in the current study.

## RESULTS

### Ulk1 over-expression in fresh human gastric cancer tissues

**Figure 1:**
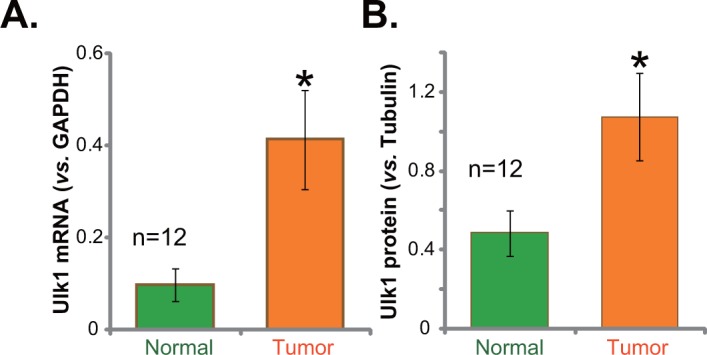
Ulk1 over-expression in fresh human gastric cancer tissues The fresh human gastric cancer tissues (“Tumor”, n=12) and the surrounding normal gastric epithelial tissues (“Normal”, n=12) were lysed; Expressions of *Ulk1* mRNA (**A**) qRT-PCR assay and Ulk1 protein (Western blotting assay) were tested. Ulk1 protein expression was quantified (**B**) *vs*. loading Tubulin, n=12. *p<0.05 *vs*. “Normal”.

We first tested expression of Ulk1 in human gastric cancer specimens. Tissue lysates were prepared from the fresh gastric cancer samples. Quantitative real-time PCR (“qRT-PCR”) assay was performed to test *Ulk1* mRNA expression in above lysates. Results in Figure 1A demonstrated that *Ulk1* mRNA level was significantly elevated in the gastric cancer tissues (“Tumor”, n=12), as compared to that in the surrounding normal gastric epithelial tissues (“Normal”, n=12) (Figure 1A). *Ulk1* mRNA level in cancer tissues was about four times higher than that in the normal tissues (Figure 1A). Western blotting assay was applied next to test Ulk1 protein expression in above tissues. Quantified blot results integrating total 12 tissue samples confirmed significant Ulk1 protein upregulation in cancer tissues (Figure 1B). Together, these results demonstrate Ulk1 over-expression in fresh human gastric cancer tissues.

### Ulk1 over-expression in human gastric cancer cells

**Figure 2:**
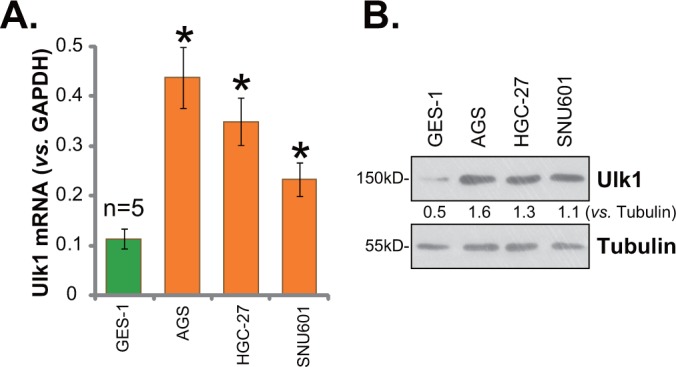
Ulk1 over-expression in human gastric cancer cells Human gastric cancer cell lines (AGS, HGC-27, and SNU601) and the GES-1 gastric mucosal epithelial cells were subjected to qRT-PCR assay **(A)** and Western blotting assay **(B)** to test Ulk1 expression. * *p*<0.05 *vs*. GES-1 cells. Experiments in this figure were repeated three times, and similar results were obtained.

Next, we tested expression of Ulk1 in human gastric cancer cells. In this study, three distinct gastric cancer cell lines, AGS, HGC-27, and SNU601, were cultured. qRT-PCR assay was again employed to test *Ulk1* mRNA expression in the cancer cell lines, and its level was compared with that in GES-1 gastric mucosal epithelial cells (non-cancerous normal cells [22]). Results in Figure 2A demonstrated that *Ulk1* mRNA level was significantly higher in the above gastric cancer cells than that in GES-1 epithelial cells. Consequently, Ulk1 protein expression was also increased in cancer cells (Figure 2B). Quantification results showed about 2-3 times higher of Ulk1 protein expression in cancer cells (*vs*. GES-1 epithelial cells, Figure 2B). Among all the tested cancer cell lines, AGS cells expressed highest level of *Ulk1* (Figure 2A and 2B). This cell line was chosen for further studies.

### Ulk1 shRNA knockdown inhibits gastric cancer cell survival

**Figure 3:**
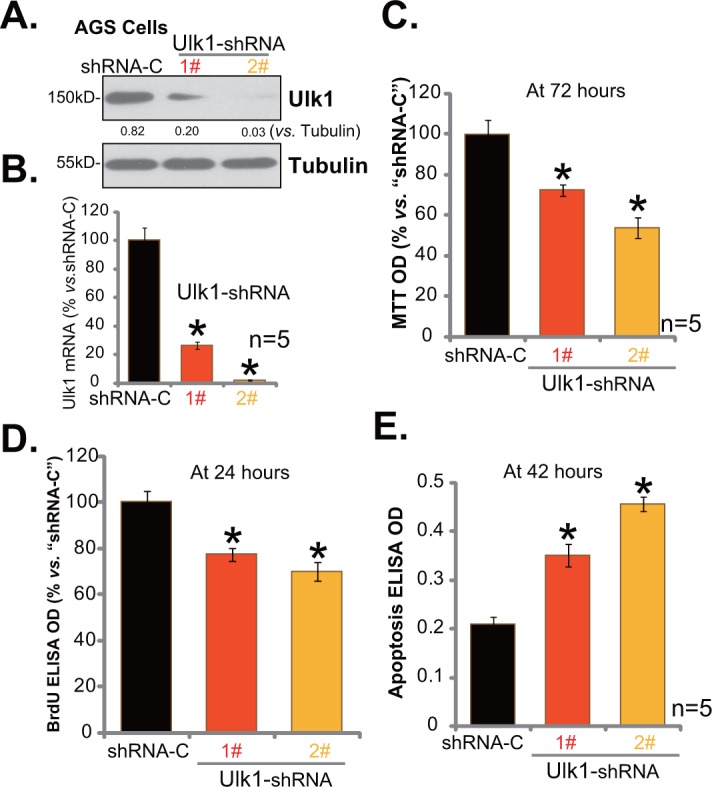
Ulk1 shRNA knockdown inhibits gastric cancer cell survival Expressions of Ulk1 protein **(A)** and *Ulk1* mRNA **(B)** in AGS cells with lentiviral Ulk1 shRNA (“1#” or “2#”) or scramble control shRNA (“shRNA-C”) were shown. Above cells were also subjected to MTT assay **(C)**, BrdU ELISA assay **(D)** and Histone DNA ELISA assay **(E)** to test cell survival, proliferation and apoptosis, respectively. For these assays, exact same number of viable cells with listed shRNA was plated initially (At 0 hour). *p<0.05 *vs*. “shRNA-C” cells. Experiments in this figure were repeated four times, and similar results were obtained.

In order to study the function of Ulk1 in gastric cancer cell survival, shRNA strategy was employed. As demonstrated, two distant lentiviral Ulk1 shRNAs (“1#” and “2#”), with non-overlapping targeting sequence, were applied here. As shown in Figure 3A and 3B, the two Ulk1 shRNAs (“1#” and “2#”) both efficiently downregulated Ulk1 in AGS gastric cancer cells. Ulk1 protein (Figure 3A) and *Ulk1* mRNA (Figure 3B) were both decreased after infection of the shRNA. Notably, Ulk1 shRNA-2# was significantly more efficient than Ulk1 shRNA-1# in silencing Ulk1 (Figure 3A and 3B). MTT assay was applied next to test survival of above cells. Results in Figure 3C demonstrated that Ulk1 shRNA knockdown decreased MTT viability OD of AGS cells, indicating an anti-survival activity by Ulk1 shRNA. Meanwhile, AGS cell proliferation, tested by the BrdU ELISA assay, was also inhibited after Ulk1 knockdown (Figure 3D). On the other hand, basal cell apoptosis intensity was enhanced following Ulk1 depletion in AGS cells (Figure 3E). Cell apoptosis was quantified via the Histone DNA ELISA assay (Figure 3E). Notably, Ulk1 shRNA-2# was more dramatic than Ulk1 shRNA-1# in inhibiting AGS cell survival (Figure 3C), proliferation (Figure 3D), and promoting cell apoptosis (Figure 3E).

### Exogenous over-expression of Ulk1 promotes gastric cancer cell survival

**Figure 4:**
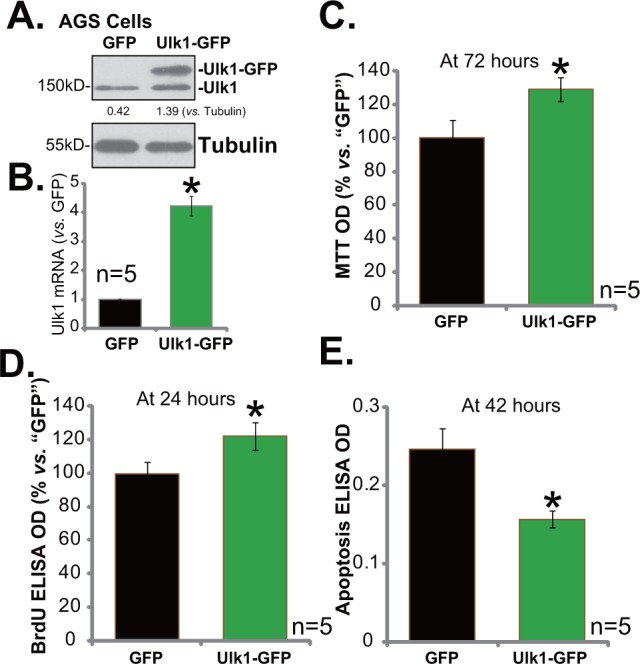
Exogenous over-expression of Ulk1 promotes gastric cancer cell survival Expressions of Ulk1 protein **(A)** and *Ulk1* mRNA **(B)** in AGS cells with exogenous Ulk1 (GFP-tagged) or empty vector (pSuper-puro-GFP, “GFP”) were shown. Above cells were also subjected to MTT assay **(C)**, BrdU ELISA assay **(D)** and Histone DNA ELISA assay **(E)** to test cell survival, proliferation and apoptosis, respectively. For the assays, exact same number of viable cells with Ulk1-GFP or empty vector was plated initially (At 0 hour). *p<0.05 *vs*. “GFP” cells. Experiments in this figure were repeated three times, and similar results were obtained.

Based on the above results, it is speculated that Ulk1 over-expression might possibly promote gastric cancer cell survival and proliferation. Thus, an Ulk1 expression vector, tagged with green fluorescence protein (GFP), was constructed. This construct was transfected to AGS cells. Western blotting assay results in Figure 4A confirmed expression of Ulk1-GFP in the transfected stable AGS cells. *Ulk1* mRNA level was also remarkably elevated in the Ulk1-GFP-expressing cells (Figure 4B). Significantly, exogenous over-expression of Ulk1 indeed promoted AGS cell survival (Figure 4C, tested by MTT assay) and proliferation (Figure 4D, tested by the BrdU ELISA assay). Meanwhile, basal apoptosis intensity, or apoptosis ELISA OD, was decreased following Ulk1 over-expression in AGS cells (Figure 4E). Therefore, exogenous over-expression of Ulk1 promotes AGS cell survival.

### Ulk1 over-expression in gastric cancer correlates with patients’ T classification and cancer relapse

To further test Ulk1 expression, we examined its level in paraffin-embedded tissues via immunohistochemistry (IHC) staining. A total of 145 patients with gastric cancer (112 males and 33 females; mean age 58.9 years, Table 1), administrated at author's hospital, were enrolled. Among all the patients, 25, 34 and 86 patients were at stage I, II and III, respectively (based on AJCC TNM staging system). Their paraffin-embedded cancer tissues were tested.

**Table 1:** Correlation between Ulk1 expression and clinical features of gastric cancer

Patients characteristics	Ulk1(low)		Ulk1(high)		P value
	cases	%	cases	%	
**Age (years)**					0.653
≤60	16	20%	64	80%	
>60	15	23.10%	50	76.90%	
**Sex**					0.979
Male	24	21.40%	88	78.60%	
Female	7	21.20%	26	78.80%	
**T classification**					0.014*
T1	8	50.00%	8	50.00%	
T2	7	23.30%	23	76.70%	
T3	1	6.30%	15	93.80%	
T4a	15	18.10%	68	81.90%	
**N classification**					0.737
N0	12	25.50%	35	74.50%	
N1	6	20.70%	23	79.30%	
N2	8	22.20%	28	77.80%	
N3	5	15.20%	28	84.80%	
**Stage**					0.227
I	8	32.00%	17	68%	
II	8	23.50%	26	76.50%	
III	15	17.40%	71	76.50%	
**Lauren classification**					0.224
Intestinal	20	24.40%	62	75.60%	
Mixed	5	29.40%	12	70.60%	
Diffuse	6	13.00%	40	87.00%	
**N classification**					0.56
Poor	7	15.60%	38	84.40%	
M-P	18	25.00%	54	75.00%	
M	6	23.10%	20	76.90%	
W-M	0	0%	2	100%	
**Vessel invasion**					0.15
No	26	24.30%	81	75.70%	
Yes	5	13.20%	33	86.80%	
**Relapse**					0.007*
No	27	27.80%	70	72.20%	
Yes	4	8.30%	44	91.70%	

Ulk1 was stained as buffy color in the cytoplasm. High expression of Ulk1 correlated with T classification (P<0.05) and cancer relapse (P<0.05). Ulk1 expression did not correlate with age, sex, N classification, stage, Lauren classification, differentiation, histological grade, and Vessel invasion (P>0.05).

**Figure 5:**
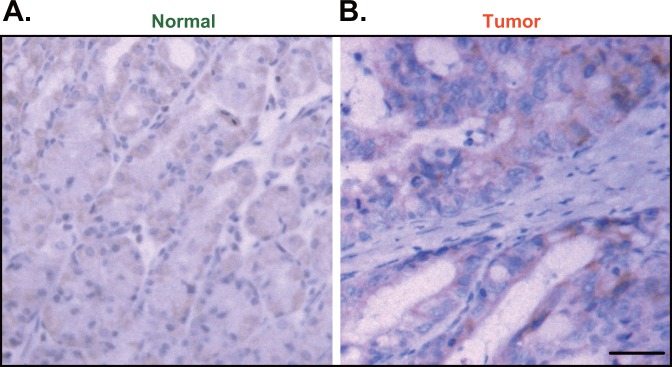
Immunohistochemistry (IHC) staining of Ulk1 in paraffin-embedded tissues Representative Ulk1 IHC images in paraffin-embedded gastric cancer tissues (“Tumor”, **B**) and surrounding normal epithelial tissues (“Normal”, **A**) (Bar=100 μm).

The IHC staining assay results showed that Ulk1 was mainly expressed in the cytoplasm of cancer cells (Figure 5A and 5B). Again, Ulk1 IHC intensity was significantly higher in the cancer tissues (“Tumor”, Figure 5A) than that in the surrounding normal epithelial tissues (“Normal”, Figure 5B). As summarized in Table 1, the majority (114 out of 145) gastric cancers showed high Ulk1 expression, and the other 31 patients showed low Ulk1 expression. Table 1 demonstrated the correlation between Ulk1 expression and patients’ clinic-pathological characteristics. Among all the clinic features, high Ulk1 expression was significantly correlated with T classification (P=0.014) and cancer relapse (P=0.007). In accordance with chi-square test, the Ulk1 overexpressed cancers have higher recurrence rate compared with that with low level of Ulk1. Notably, high Ulk1 expression did not correlate with age, sex, N classification, stage, Lauren classification, differentiation, histological grade, and Vessel invasion (P > 0.05) (Table 1). These results suggest that high Ulk1 expression in gastric cancer is correlated with patients’ T classification and cancer relapse.

## DISCUSSION

Although sustained and intensified autophagy could promote cell death (“autophagic cell death”), minor and moderate autophagy, seen in the majority of occasions, could promote cell survival [12–15]. In those cases, cellular components are digested to provide nutrients and energies for cell survival [12–15]. This process shall help cells to survival under conditions like hypoxia and nutrient/growth factor deprivation, which are frequently happening in cancer cells [23–25].

The process of autophagy starts with formation of the double membrane vesicles (autophagosomes) in the cytoplasm, which degrade cytoplasmic materials by acidic lysosomal hydrolases [26]. Ulk1 is vital for autophagy initiation, via integrating signals from upstream sensors, including mTOR (mammalian target of rapamycin) and AMPK (AMP-activated protein kinase) [18]. For example, AMPK phosphorylates and activates Ulk1 to initiate autophagy [18, 21]. On the other hand, mTOR phosphorylates Ulk1 at different sites of AMPK, and inhibits Ulk1 activity [18, 21] to shut down autophagy. Recent studies have also proposed other mechanisms of Ulk1 in promoting cell autophagy and also mitophagy [10].

Our *in vitro* studies showed that knockdown of Ulk1 by targeted shRNAs inhibited AGS cell survival and proliferation, but inducing cell apoptosis. On the other hand, exogenous over-expression of Ulk1 promoted AGS cell survival and proliferation. These evidences implied that high Ulk1 expression in human gastric cancer cells should be pro-survival.

Studies testing Ulk1 expression in cancer cells have not been able to reach consistent conclusion. Pike *et al*., showed that transcriptional up-regulation of Ulk1 is important for cancer cell survival [27], and that loss of Ulk1 could promote necrotic cancer cell death. Same study implied that *Ulk1* mRNA upregulation is associated with poor prognosis of breast cancer [27]. On the other hand, Tang *et al*., suggested that low Ulk1 expression is associated with progression of certain breast cancers [28]. We showed that Ulk1 (mRNA and protein) expression was significantly elevated in 12 different fresh human gastric cancer tissues. Meanwhile, Ulk1 over-expression was also noticed in several gastric cancer cell lines. IHC staining assay results further confirmed that 114 gastric cancer tissues showed high Ulk1 expression, and only 31 patients showed low Ulk1 expression. Importantly, high Ulk1 expression in gastric cancer was significantly correlated with patients’ T classification and cancer relapse. These *in vitro* and *in vivo* evidences suggest that over-expressed Ulk1 could be an important oncotarget protein for human gastric cancer. Further studies will be needed to enlarge the sample size to further support this conclusion. Patients’ survival data should also be followed.

## MATERIALS AND METHODS

### Cell culture

The established human gastric cancer cell lines, AGS, HGC-27, and SNU601, as well as the GES-1 gastric mucosal epithelial cells were obtained from the Cell Bank of CAS (Shanghai, China) at Dec 2013. Cells were maintained in RPMI/DMEM medium with 10% fetal bovine serum (FBS). The verification of each single cell line was described previously [29]. The reagents for cell culture were from Gibco (Shanghai, China).

### Reagents and antibodies

Puromycin was provided by Sigma (Shanghai, China). All the antibodies in this study were purchased from Cell Signaling Tech (Danvers, MA).

### Fresh human gastric cancer tissues

Surgery-isolated human gastric cancer tissues were washed. Tumor tissues and surrounding normal tissues were separated. A total of twelve patients with gastric cancer were enrolled (8 male, 4 female, 42-67 years old). These patients received no other therapies prior to the surgery. Fresh tissues were stored in liquid nitrogen. Tissue lysis buffer (Biyuntian, Wuxi, China) was applied to homogenate the tissues [30–33]. The protocols using human samples were approved by the Internal Review Board (IRB) and Ethics Board (EB) of authors’ institutions. The written-informed consent was obtained each and every participant. All studies were conducted according to the principles expressed in the Declaration of Helsinki and national/international guidelines.

### Immunohistochemistry (IHC) staining

A total of 145 gastric cancer tissues (paraffin-embedded), collected from Dec 2012-Dec 2013, were analyzed via IHC analysis. As described [34, 35], the staining was performed on cryostat sections (4 μm) of fixed human gastric cancer tissues (or the surrounding normal tissues). Primary antibody (anti-Ulk1, 1:50) and horseradish peroxidase (HRP)-coupled secondary antibody (Santa Cruz) were added to the tissue slides. The peroxidase activity was visualized via the 3-amino-9-ethyl-carbazol (AEC) and MAYER solutions (Merck). Tissue samples with rich cells and easily observable spots were considered valid, where as those with folded slices, coloring failure, and spots without cells were considered invalid. The degree of immuno-staining was reviewed and scored independently by two observers based on the intensity of staining. Staining intensity was graded according to the following criteria: 0 (no staining), 1 (weak staining =light yellow), 2 (moderate staining= yellow brown), and 3 (strong staining= brown). Moderate and strong staining were utilized to define tumors with high Ulk1 expression, and no and weak staining were used to indicate low Ulk1 expression.

### RNA extraction and real-time PCR

As reported [29, 34, 36], total RNA from cell/tissue lysates was obtained via the Trizol reagents (Invitrogen). Quantitative Real Time-PCR (“qRT-PCR”) assay was performed. The PCR reaction mixture contained SYBR Master Mix (Applied Bio-system), 0.5 μg RNA and 100 nM primers. The ABI Prism 7500 Fast Real-Time PCR system (Foster City, CA) was employed for PCR assay. The primers for *Ulk1* mRNA and *GAPDH* mRNA were described previously [27]. Melt curve analysis was tested to analyze product melting temperature. *GAPDH* was always analyzed as the reference gene. The 2^−ΔΔ*C*t^ method was applied to quantify targeted expression change within samples [34, 36].

### Western blotting assay

As previously described [29, 32, 37, 38], protein lysates (30 μg each lane) were separated by SDS-page gel (10%), and were transferred to the polyvinylidene difluoride (PVDF) membranes (Millipore). After blocking, membranes were incubated with specific primary and secondary antibodies. Enhanced chemiluminescence (ECL) reagents (Amersham, Shanghai, China) were applied for detection of the band. ImageJ software was utilized to quantify total gray of each band, and its level was normalized to Tubulin (loading).

### MTT assay

Cell survival was tested by the routine 3-(4,5-dimethylthiazol-2-yl)-2,5-diphenyltetrazolium bromide (MTT) assay as described previously [35].

### BrdU ELISA assay of cell proliferation

Cells with applied treatment were incubated with BrdU (10 μM, Cell Signaling Tech, Shanghai, China). BrdU incorporation was determined in the ELISA format [33]. ELISA OD was utilized as an indicator or cell proliferation.

### Histone DNA ELISA assay

Quantification of cell apoptosis by the Histone DNA ELISA assay was reported in our previous studies [38, 32].

### Ulk1 shRNA knockdown

The two distinct Ulk1 lentiviral shRNAs (packed in GV428 vector), targeting non-overlapping sequence of human *Ulk1*, were designed by the Genepharm (Shanghai, China). These two shRNAs were added directly to AGS cells (with 50-60% of confluence). After 24 hours, virus-containing medium was replaced with fresh complete medium. Stable AGS cells were then selected by puromycin (2.5 μg/mL, Sigma) for another 3 days. Expression of Ulk1 in the stable cells was verified by Western blotting assay and qRT-PCR assay.

### Exogenous Ulk1 over-expression

The Ulk1 cDNA, provided again by Genepharm, was cloned into the pSuper-puro-GFP vector [29], which was transfected into AGS cells via Lipofectamine 2000 (Invitrogen). After 24 hours, cells were re-plated on selection medium containing puromycin (2.5 μg/mL, Sigma). Expression of Ulk1 (GFP-tagged) in the resulting cells was tested by Western blotting assay and qRT-PCR assay.

### Statistical analyses

The SPSS software 15.0 version was used for statistical analysis. The χ2 test was applied to analyze the relationship between Ulk1 expression and patients’ clinical features. P values for all comparisons were two-tailed. Statistical significance was defined as P <0.05 for all tests.

.
